# The proteolytic system of lactic acid bacteria revisited: a genomic comparison

**DOI:** 10.1186/1471-2164-11-36

**Published:** 2010-01-15

**Authors:** Mengjin Liu, Jumamurat R Bayjanov, Bernadet Renckens, Arjen Nauta, Roland J Siezen

**Affiliations:** 1 Centre for Molecular and Biomolecular Informatics, Radboud University Medical Centre, Nijmegen, the Netherlands; 2 FrieslandCampina Research, Deventer, the Netherlands; 3 NIZO food research, Ede, the Netherlands; 4 TI Food and Nutrition, Wageningen, the Netherlands

## Abstract

**Background:**

Lactic acid bacteria (LAB) are a group of gram-positive, lactic acid producing Firmicutes. They have been extensively used in food fermentations, including the production of various dairy products. The proteolytic system of LAB converts proteins to peptides and then to amino acids, which is essential for bacterial growth and also contributes significantly to flavor compounds as end-products. Recent developments in high-throughput genome sequencing and comparative genomics hybridization arrays provide us with opportunities to explore the diversity of the proteolytic system in various LAB strains.

**Results:**

We performed a genome-wide comparative genomics analysis of proteolytic system components, including cell-wall bound proteinase, peptide transporters and peptidases, in 22 sequenced LAB strains. The peptidase families PepP/PepQ/PepM, PepD and PepI/PepR/PepL are described as examples of our *in silico *approach to refine the distinction of subfamilies with different enzymatic activities. Comparison of protein 3D structures of proline peptidases PepI/PepR/PepL and esterase A allowed identification of a conserved core structure, which was then used to improve phylogenetic analysis and functional annotation within this protein superfamily.

The diversity of proteolytic system components in 39 *Lactococcus lactis *strains was explored using pangenome comparative genome hybridization analysis. Variations were observed in the proteinase PrtP and its maturation protein PrtM, in one of the Opp transport systems and in several peptidases between strains from different *Lactococcus *subspecies or from different origin.

**Conclusions:**

The improved functional annotation of the proteolytic system components provides an excellent framework for future experimental validations of predicted enzymatic activities. The genome sequence data can be coupled to other "omics" data e.g. transcriptomics and metabolomics for prediction of proteolytic and flavor-forming potential of LAB strains. Such an integrated approach can be used to tune the strain selection process in food fermentations.

## Background

Lactic acid bacteria (LAB) have been used for centuries as starter or adjunct cultures in dairy fermentations. The breakdown of milk proteins (proteolysis) by LAB plays an important role in generating peptides and amino acids for bacterial growth and in the formation of metabolites that contribute to flavor formation of fermented products. The proteolytic system of LAB comprises three major components: (i) cell-wall bound proteinase that initiates the degradation of extracellular casein (milk protein) into oligopeptides, (ii) peptide transporters that take up the peptides into the cell, and (iii) various intracellular peptidases that degrade the peptides into shorter peptides and amino acids. In particular, as caseins are rich in proline, LAB have numerous proline peptidases for degrading proline-rich peptides [1-3]. Amino acids can be further converted into various flavor compounds, such as aldehydes, alcohols and esters [4].

Several reviews have described the proteolytic system of LAB with respect to their biochemical and genetic aspects [1,5-8]. In the past ten years, however, many LAB genomes have been sequenced, which allows a thorough comparative analysis of their proteolytic systems at a genome scale. In a preliminary study, we described a comparative analysis of cell-wall-bound proteinase and various peptidases from 13 fully or incompletely sequenced LAB which were publicly available in May 2006 [9]. More recently, over ten additional LAB genomes have become publicly available. These include 8 LAB strains from the Joint Genome Institute and the LAB Genome Consortium [10], the model laboratory strain *Lactococcus lactis *subsp. *cremoris *MG1363 [11], a *Lactobacillus helveticus *strain [12] which is known for its proteolytic capacity as an adjunct culture in cheese, and the probiotic strain *Lactobacillus rhamnosus *GG [13]. Furthermore, a recent comparative genome hybridization (CGH) analysis of 39 *L. lactis *strains [14] provides opportunities to explore the diversity of the proteolytic system within the same species.

In this study, we systematically explored the diversity of the cell-wall bound proteinase, the peptidases and the peptide transporters in twenty-two completely sequenced LAB strains. The distinctions between subgroups in large peptidase families such as the PepP/PepQ/PepM family, the PepD family and the PepI/PepR/PepL family are described in detail as examples. The PepI/PepR/PepL family was compared with the EstA family of esterases, the key enzyme for synthesizing various ester flavors [4,15], since the members of these two families share sequence and structure homology. Furthermore, the results from comparative genomics analysis were used to explore the diversity of members of the proteolytic system in 39 *Lactococcus lactis *strains by pangenome CGH analysis [14].

## Methods

### Comparative genome analyses and orthologous groups identification

Complete genome sequences of LAB were obtained from the NCBI microbial genome database http://www.ncbi.nlm.nih.gov/genomes/lproks.cgi. The genomes include: *Lactobacillus acidophilus *NCFM (abbreviation LAC, accession code CP000033), *Lactobacillus johnsonii *NCC 533 (LJO, AE017198), *Lactobacillus gasseri *ATCC 33323 (LGA, CP000413), *Lactobacillus delbrueckii *subsp. *bulgaricus *ATCC 11842 (LDB, CR954253), *Lactobacillus delbrueckii *subsp. *bulgaricus *ATCC BAA365 (LBU, CP000412), *Lactobacillus plantarum *WCFS1 (LPL, AL935263), *Lactobacillus brevis *ATCC 367 (LBE, CP000416), *Lactobacillus sakei *23 K (LSK, CR936503), *Lactobacillus salivarius *UCC118 (LSL, CP000233), *Oenococcus oeni *PSU1 (OOE, CP000411), *Pediococcus pentosaceus *ATCC 25745 (PPE, CP000422), *Leuconostoc mesenteroides *ATCC 8293 (LME, CP000414), *Lactobacillus casei *ATCC 334 (LCA, CP000423), *Lactococcus lactis *subsp. *lactis *IL1403 (LLX, AE005176), *Lactococcus lactis *subsp. *cremoris *MG1363 (LLM, AM406671), *Lactococcus lactis *subsp. *cremoris *SK11 (LLA, CP000425), *Streptococcus thermophilus *CNRZ1066 (STH, CP000024), *Streptococcus thermophilus *LMG18311 (STU, CP000023), *Streptococcus thermophilus *LMD9 (STM, CP000419), *Lactobacillus reuteri *F275 (LRF, CP000705), *Lactobacillus helveticus *DPC 4571 (LHE, CP000517) and *Lactobacillus rhamnosus *GG (LRH, FM179322). Incomplete genome sequences of *Lactococcus lactis *subsp. *lactis *strains KF147 and KF282 [16] were additionally used for analysis of *L. lactis *strain diversity by pangenome CGH analysis [14].

Protein sequences of experimentally verified proteolytic system members, i.e. cell-wall bound proteinase, various peptidases and peptide transporters, were derived from the non-redundant protein database Uniprot http://www.uniprot.org/[17]. These sequences were used to perform a BLASTP [18] search against all LAB genomes. The corresponding Hidden Markov Models (HMMs) of each protein family were obtained from the Pfam database [19] and utilized to search for homologous genes using the HMMER 2.3.2 package http://hmmer.janelia.org/. The homologous sequences of each proteinase, peptidase and peptide transporter were collected on basis of the BLAST and HMM search results and redundancies were removed. Orthologous groups (subfamilies) were identified by an in-house developed method [4,20]. Multiple sequence alignments (MSA) were generated for each homologous group using MUSCLE [21]. Bootstrapped (n = 1000) neighbor-joining family trees were constructed with ClustalW [22]. The trees were visualized in LOFT [23] and orthologous groups were identified. The gene contexts were analyzed using the ERGO Bioinformatics Suite [24] to improve ortholog prediction when necessary.

### 3D structure alignment

Peptidases PepI/PepR/PepL and esterase EstA belong to the same protein superfamily, but they possess different functionalities. In order to identify substrate specificity of each protein subfamily, a comparison of known protein 3D structures was carried out. As described above, protein sequences of experimentally characterized peptidases PepI, PepR, and PepL, together with EstA esterases were used to search against all the sequenced LAB genomes and other prokaryote genomes in the NCBI database by BLASTP [18]. Moreover, the HMM of the protein α/β hydrolase fold PF00561 from the Pfam database [19], to which PepI/PepR/PepL and EstA belong, was used to search against LAB genomes. Homologs of both PepI/PepR/PepL and EstA families were collected. Similarly, the protein sequences of experimentally verified PepI/PepR/PepL and EstA members were used for BLAST searches against the PDB database http://www.rcsb.org/pdb/[25]. The protein sequences, as well as the 3D structures of the best BLAST hits were collected. Other proteins with similar structures were retrieved by the Dali server http://ekhidna.biocenter.helsinki.fi/dali_server/ using the protein structures of the BLAST hits as input.

The retrieved 3D structures of the proteins used as templates in this study are: the tricorn interacting factor F1 with proline iminopeptidase (PIP) activity from *Thermoplasma acidophilum *(PDB ID: 1MTZ), proline iminopeptidases from *Xanthomonas campestris *pv. *citri *(PDB ID: 1AZW) and *Serratia marcescens *(PDB ID: 1WM1) as members of PepI/R/L subfamilies, and the esterase (PDB ID: 2UZ0) from *Streptococcus pneumoniae *which belongs to the EstA subfamily. These 3D structures were superimposed and visualized by the YASARA program (version 6.813, http://www.yasara.org/). Conserved superimposable regions (core regions) of the catalytic domain were identified based on the 3D-structure alignment, and these consisted of 4 discontinuous sequence segments that are connected by loops of variable structure.

The amino acid sequences of the four core region segments were aligned with MUSCLE or ClustalW as described [26]. The alignments were manually curated for ambiguously aligned sequences compared to the 3D-structure alignment. Sequences with more than 90% identity were removed. Finally, a MSA was constructed based on concatenated alignments of all the curated local alignments of the core regions [see Additional File 1]. A bootstrapped (n = 1000) neighbor-joining tree on basis of the MSA was constructed and orthologous groups, so-called subfamilies, were identified automatically by LOFT.

### Pangenome CGH diversity analysis

Comparative genome hybridization (CGH) data of 39 *L. lactis *strains was acquired from pangenome arrays [14]. The pangenome array was constructed on basis of publicly available complete genome sequences of *L. lactis *subsp. *lactis *IL1403, *L. lactis *subsp. *cremoris *SK11, and incomplete genome sequences of *L. lactis *strains KF147 and KF282, as described by Bayjanov et al. [14]. The CGH data used in this study can be found under the accession number GSE12638 in the NCBI GEO (NCBI **G**ene **E**xpression **O**mnibus) database http://www.ncbi.nlm.nih.gov/projects/geo/query/acc.cgi?acc=GSE12638.

The genes encoding predicted proteolytic system components of the three sequenced *L. lactis *strains were used to query the database containing pangenome CGH data. We obtained a statistical score of the hybridization signal for each gene from the reference strains against 39 *L. lactis *strains. A cut-off value 5.5 was used to assign presence or absence of every gene from the proteolytic system in query strains, as described by Bayjanov et al. [14]. In most cases, a gene is regarded present in a specific strain if it has a maximum score higher than 5.5 [14].

## Results

### The distribution of proteolytic system components in sequenced LAB genomes

**Figure 1:**
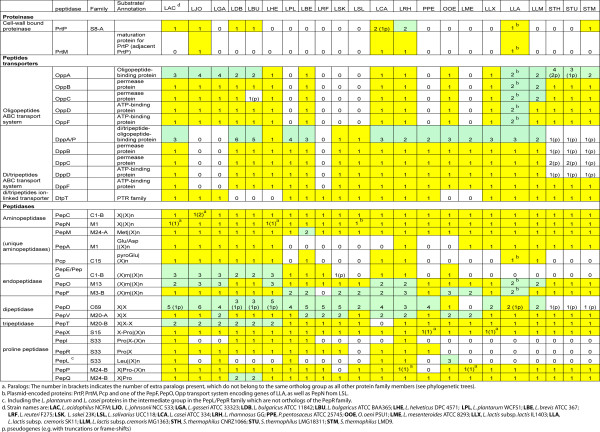
**Distribution of proteinase, peptide transporters and peptidases of the proteolytic system in LAB**. The number of identified genes is indicated. MEROPS families are indicated for proteinase and peptidases. Color shading shows absence of a gene (white), a single gene (yellow) or multiple genes (green). The GI codes of the genes can be found in Additional File 2.

An overview of the distribution of components of the proteolytic system identified in 22 completely sequenced LAB is given in Figure 1. A detailed list of genes with GI codes can be found in Additional File 2. The number of genes encoding putative members of each proteinase, peptide transporter and peptidase subfamily are shown.

The LAB genomes in the *L. acidophilus *group [4], including *L. acidophilus, L. johnsonii, L. gasseri, L. bulgaricus*, and *L. helveticus *strains, encode a relatively higher number and variety of proteolytic system components. Some enzymes are only found in a few LAB strains, such as the cell-wall bound proteinase (PrtP). PrtP was only found on the chromosome of *L. acidophilus, L. johnsonii, L. bulgaricus, L. casei*, *L. rhamnosus *and *S. thermophilus *strain LMD9, as well as on the plasmid of *L. lactis *subsp. *cremoris *SK11 [27]. Members of both the PepE/PepG (endopeptidases) and PepI/PepR/PepL (proline peptidases) superfamilies are absent in lactococci and streptococci. On the other hand, many of the peptidases seem to be essential for bacterial growth or survival as they are encoded in all LAB genomes. For instance, aminopeptidases PepC, PepN, and PepM, and proline peptidases PepX and PepQ are present in all genomes, usually with one gene per genome. Some LAB genomes have two peptidase homologs, possibly with the same function (shown in brackets in Figure 1), e.g. two PepC homologs (GI codes: 42518641 and 42518638) in *L. johnsonii*. Other essential peptidases (found in all LAB genomes) such as endopeptidase PepO and dipeptidase PepV are encoded by multiple paralogous genes.

*L. acidophilus*, *L. brevis*, *L. casei*, *L. rhamnosus *and *L. lactis *strains possess all three known LAB peptide transport systems, i.e. the di/tripeptide Dpp and DtpT systems and the oligopeptide Opp system [5]. In contrast, *L. reuteri *strain only has one functional peptide transport system, the DtpT system. Several peptide transporters or peptidases fall into larger protein superfamilies. Examples are (i) the oligopeptide-binding protein OppA and di/tripeptide-binding proteins DppA/DppP in the same peptide-binding protein family, (ii) aminopeptidase PepC together with endopeptidases PepE and PepG belonging to MEROPS peptidase family C1-B, (iii) proline peptidases PepI, PepR and PepL belonging to MEROPS family S33, and (iv) aminopeptidase PepM together with proline peptidases PepP and PepQ belonging to MEROPS family M24 (Figure 1). Protein members in those large superfamilies share high sequence similarity, and cannot always be distinguished by simple BLAST sequence homology searches. Using a comparative genomics approach, the large protein families can be divided into subfamilies with putatively different substrate specificities. For example, the aminopeptidase PepC subfamily can be clearly distinguished from the endopeptidase PepE/PepG subfamily as they are separated into distinct groups in a superfamily tree [9]. In other cases, such as the endopeptidase PepF family, several distinct subgroups can be distinguished but the difference in specificity between the subgroups is still unclear [see Additional File 3].

Three large peptidase families (PepP/PepQ/PepM, PepD and PepI/PepR/PepL) will be discussed in detail in the following sections.

### Subfamilies of peptidase family PepP/PepQ/PepM

PepP, PepQ and PepM belong to the MEROPS peptidase family M24 which requires metal ions for catalytic activity. PepM is a methionyl aminopeptidase cleaving N-terminal methionine from proteins. PepP is a member of the proline peptidases which cleave off any N-terminal amino acid linked to proline in an oligopeptide. PepQ is also a proline peptidase, however specific for Xaa-Pro dipeptides, where Xaa represents any amino acid (Figure 1)

**Figure 2:**
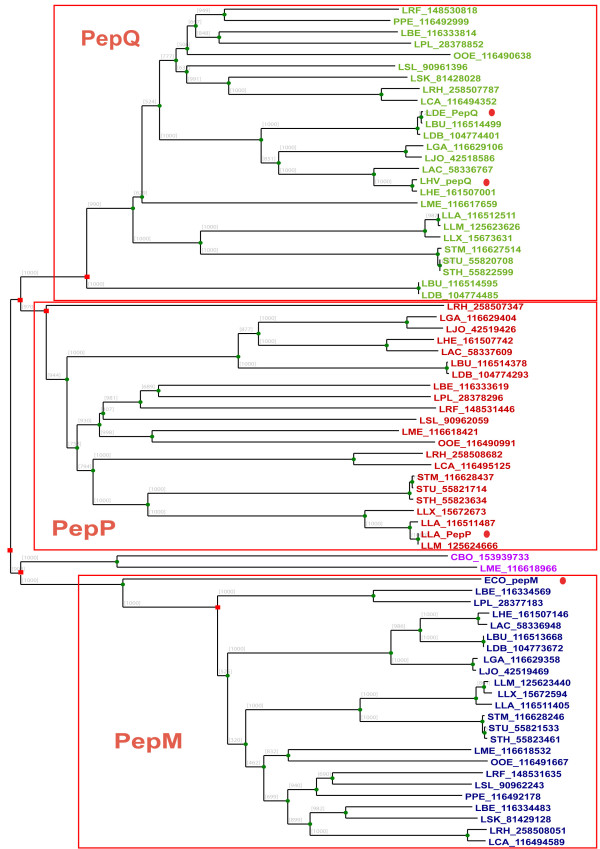
**Superfamily tree of PepP/PepQ/PepM members in LAB**. Genome abbreviations can be found in "Methods". For each gene, the organism abbreviations are followed by GI codes. Homologs from two non-LAB strains are also included, CBO for *Clostridium botulinum *F str. Langeland and ECO for *E. coli*. Experimentally characterized genes are highlighted by the red dots. Green circles represent the speciation events, and red squares represent duplication events.

Our phylogenetic analysis shows that PepP, PepQ and PepM are separated into three distinct subgroups in accordance with the known different substrate specificities of each peptidase (Figure 2). PepP and PepQ seem to be more closely related than PepM on the basis of the family tree, which is in agreement with the differences in their catalytic activities. Bacterial PepM is an aminopeptidase belonging to subfamily M24A which usually requires cobalt ions for catalysis, while PepP and PepQ as proline peptidases belong to the subfamily M24B which prefers manganese [1].

In the PepP subgroup, one gene is found in each LAB genome except in *L. sakei *and *Pediococcus pentosaceus*. The absence of the *pepP *genes in both genomes is very likely due to a gene loss event. The family tree also includes an experimentally verified *pepP *gene from *L. lactis *whose protein product has been purified and characterized [28]. Moreover, LAB-derived *pepP *genes are always flanked on the chromosome by a gene encoding an elongation factor for protein translation. The conserved gene context of *pepP *among LAB genomes is consistent with the putative important physiological role of PepP in protein maturation, as suggested by Matos et al. [28].

Genes from the PepQ cluster are distributed equally in all LAB genomes, generally as one copy per genome. However, the *L*. *delbrueckii bulgaricus *strains have two *pepQ *paralogs. One paralog is clustered with the other orthologs of LAB, whereas the second paralog is located in a separate cluster (LBU_116514595 and LDB_104774485). This might be the result of an ancient duplication (Figure 2) or horizontal gene transfer (HGT) event. Rantanen et al. suggested that the second paralogous *pepQ *of *L. bulgaricus *is a cryptic gene [29]. Experimentally characterized *pepQ *genes from *L. delbrueckii bulgaricus *[30] and *L. helveticus *(GI: 3282339) are added and highlighted in the tree, supporting the annotation of the subgroups.

In the aminopeptidase PepM subgroup, *L. brevis *has an extra paralogous gene, which clusters together with the *L. plantarum pepM *gene. Gene context analysis suggests that *pepM *genes in all *Lactobacillus *strains share the same neighbor genes, except the *pepM *gene from *L. plantarum *and both the paralogs from *L. brevis*. One of the *L. brevis pepM *genes (LBE_116334483) is located in the same operon as a transposase. Based on the protein family tree, we hypothesize that an extra *pepM *gene was acquired first in the ancestor of *L. brevis *and *L. plantarum*, after which one gene was lost from *L. plantarum*. The *L. plantarum pepM *gene (LPL_28377183) is flanked by a methionine metabolism related operon (*cysK_cblB/cglB_cysE*). Therefore, the *pepM *gene in *L. plantarum *may have a broader function, probably utilizing proteins and peptides as methionine pool, in addition to the classic PepM function for N-terminal maturation of proteins.

One gene from *Leuconostoc mesenteroides *(LME_116618966) is located as an intermediate between the PepP/PepQ and PepM subfamilies. It shares higher sequence homology with a putative *pepP *gene from *Clostridium botulinum *(Figure 2) and has a phage-related gene in its neighborhood. This suggests that the *pepP *gene from *Leuconostoc mesenteroides *might be acquired from clostridia.

### Subfamilies of peptidase family PepD

**Figure 3:**
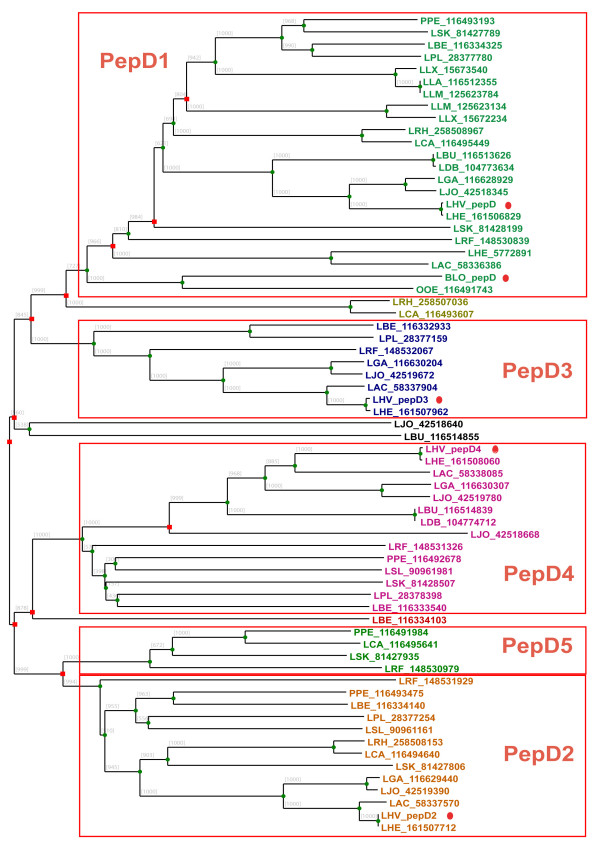
**Superfamily tree of PepD members in LAB**. PepD that is experimentally characterized from *Bifidobacterium longum *NCC2705 (BLO) [52] and *pepD *genes from *L. helveticus *CNRZ32 (LHV) analyzed by microarray [32] are indicated by the red dots. Green circles represent the speciation events, while red squares represent duplication events.

The PepD dipeptidase family has a broad specificity toward various dipeptides [1]. PepD has been purified and characterized from *L. helveticus *by Vesanto et al. [31]. The *pepD *genes are distributed heterogeneously in LAB genomes, varying from 0 to 6 paralogs. The *pepD *gene is absent in *Leuconostoc mesenteroides *and truncated in *S. thermophilus *strains, while multiple genes are mainly observed in *Lactobacillus *genomes (Figure 1). Recently, Smeianov et al. reported the expression level of four *pepD *genes from *L. helveticus *CNRZ32 by a microarray analysis [32]. Five major PepD subfamilies can be clearly distinguished based on the multiple sequence alignment (Figure 3). PepD1-4 are assigned with the names according to the four *pepD *genes from *L. helveticus *[32]. Due to the lack of experimental evidence, it is still unclear whether the substrate specificities vary between those subfamilies. Microarray analysis of *L. helveticus *has shown that *pepD1, pepD2 *and *pepD4 *were up-regulated in MRS medium compared to growth in milk, while *pepD3 *was not differentially expressed in both media [31]. It suggests that differences between subgroups of *pepD1*/*pepD2*/*pepD4 *and *pepD*3 could also be on the level of transcription regulation. Moreover, several genes are located as intermediate between the major PepD subgroups in the superfamily tree. Most of those genes have unclear origins and functions. The protein sequences of LCA_116493607 from *L. casei*, LRH_258507036 from *L. rhamnosus*, LJO_42518640 from *L. johnsonii*, and LBU_116514855 from *L. bulgaricus *have best BLASTP hits to several recently sequenced lactobacilli, such as *L. hilgardii *and *L. buchneri*, suggesting a possible duplication of the gene in a specific *Lactobacillus *group.

### 3D-structure comparison to distinguish PepI/PepR/PepL peptidases from EstA family esterases

**Figure 4:**
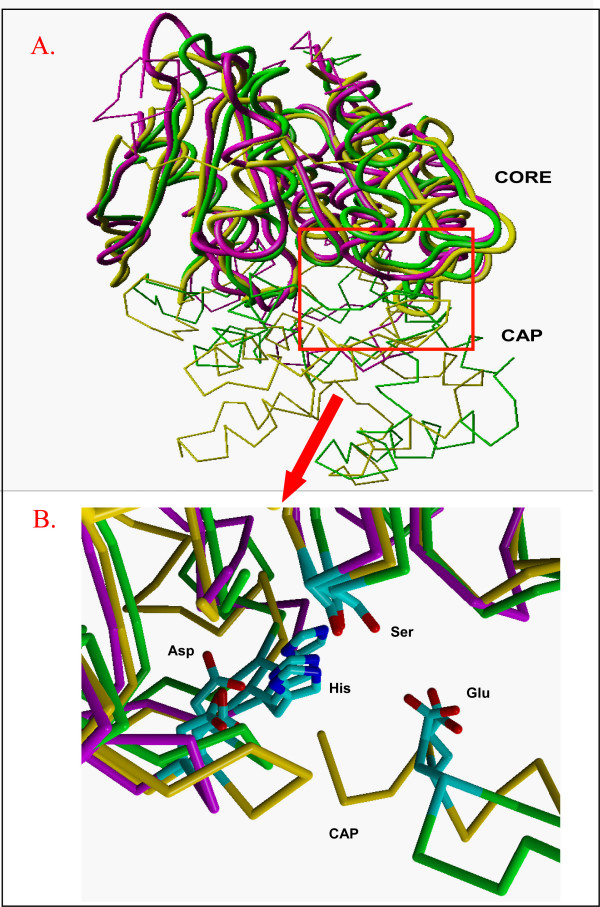
**Superposition of 3D structures of proline iminopeptidases 1WM1 (yellow) and 1MTZ (green), and esterase 2UZ0 (purple)**. The structure of 1AZW is highly similar to 1WM1 and is not shown. A) The 4 conserved structural core segments are shown as thick tubes, and the variable segments as thin sticks connecting C-alpha atoms. The variable large cap regions of the peptidases, which do not superpose, are at the bottom half of the figure. Note that the esterase has a much shorter connecting segment in this cap region. The red frame indicates the position of the active site, which is shown as the zoomed-in view in Panel B. B) The catalytic site is shown with catalytic residues Ser, His and Asp. The active site is enlarged and rotated by about 180 degrees relative to Panel A. A short stretch of the cap region in both peptidases is shown, bearing the Glu residues that interact with the positive charge of the peptide substrate N-terminus. Note that the side chains of the two Glu residues superpose very well, despite coming from different (non-superposable) parts of the cap region.

The proline iminopeptidase PepI possesses aminopeptidase activity toward N-terminal proline peptides, preferably tri-peptides, while prolinase PepR has a broad specificity for dipeptides including Pro-Xaa dipeptides [1]. The only characterized PepL is from *L. delbrueckii *subsp. *lactis *DSM7290 and it displays high specificity for di-/tri- peptides with N-terminal leucine residues [33]. Interestingly, the PepI/PepR/PepL family and the esterase EstA family belong to the same α/β hydrolase superfamily, since the BLASTP analysis of PepI/PepR/PepL members against the non-redundant protein database also retrieves homologs from the EstA family. Multiple sequence alignment (MSA) of the whole protein sequences of the homologs from those two protein families is not reliable, as large insertions and deletions are present in these sequences, and several regions of the proteins share very low sequence similarity. Therefore, we first compared the 3D structures of four representative proteins by superposition, including proline iminopeptidases from *Thermoplasma acidophilum *(PDB ID: 1MTZ) [34], *Xanthomonas campestris *pv. *citri *(PDB ID: 1AZW) [35], and *Serratia marcescens *(PDB ID: 1WM1) [36] as members from the PepI/R/L family, and an esterase A (PDB ID: 2UZ0) from *Streptococcus pneumoniae *[37] as a member from the EstA subfamily (Figure 4). The superimposed 3D structures share a highly similar catalytic domain, which displays a typical canonical α/β hydrolase topology consisting of an eight-stranded β-sheet, and have a non-conserved cap domain. Four conserved structural regions in the catalytic domain, separated by variable loops, were identified based on the structure alignment. A detailed comparison of the residues of the catalytic site and substrate-binding region can be found in Additional File 4. In contrast, the cap domain shows a large structural variation, and the esterase EstA has a much smaller cap domain than the peptidases (Figure 4). The cap regions of peptidases cover and close the substrate-binding region, allowing only the N-terminal proline of a peptide to fit into the substrate-binding pocket.

**Figure 5:**
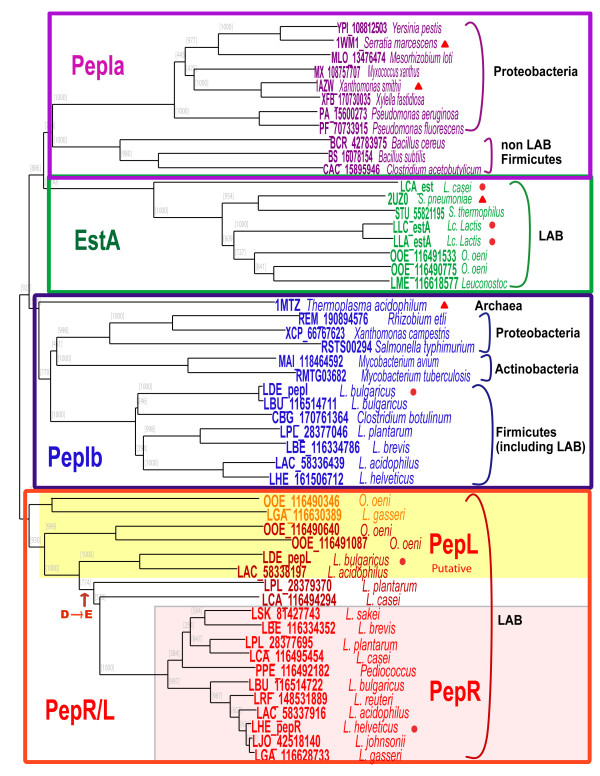
**Superfamily tree of PepI/PepR/PepL and EstA members**. Based on MSA of the concatenated sequences of the four structural core regions identified by the protein 3D structure alignment. Orthologous proteins are indicated by the same color. The PepR subgroup from LAB is shadowed in pink, and the PepL subgroup is shadowed in yellow. The bacterial phyla are indicated. Red dots indicate the experimentally characterized genes and red triangles indicate the protein 3D structures used for the analyses. The event of the substitution of catalytic residue aspartate by glutamate in the PepR subgroup is indicated in the tree.

A MSA of the concatenated sequences of the four conserved structural regions of the PepI/PepR/PepL and EstA superfamily members from various microorganisms was constructed and manually curated [See Additional File 1]. On basis of the curated MSA, a much improved superfamily tree was constructed for the PepI/PepR/PepL and EstA families, including LAB and other bacteria, as well as the reference proteins with known 3D structures (Figure 5). In this 3D alignment tree, the homologs of the superfamily can be clearly separated into four subclusters (Figure 5). The first cluster PepIa contains the proline iminopeptidases from Proteobacteria and non-LAB Firmicutes, including the ones from the known structures 1AZW and 1WM1. The second cluster contains the esterase members from LAB, including the representative structure 2UZ0 from *S. pneumoniae*. The third cluster PepIb contains proline iminopeptidases from Proteobacteria and Actinobacteria, and PepI from Firmicutes (including the ones from LAB), as well as the known structure 1MTZ from *Thermoplasma acidophilum*. The last cluster PepR/L consists of putative PepL proteins from LAB and the subgroup of prolinase PepR. Experimentally verified proteins PepR from *L. helveticus *CNRZ32 [38,39], PepI from *L. delbrueckii *subsp. *bulgaricus *CNRZ 397 [40,41], PepL from *L. delbrueckii *subsp. *lactis *DSM7290 [33] and EstA from *L. lactis *[42] and *L. casei *BL23 [43] also support this subdivision within the protein superfamily (Figure 5). Moreover, PepI from *L. helveticus *strain 53/7 has also been experimentally characterized [44].

Sequenced lactococcal, streptococcal, leuconostoc and *L. salivarius *strains lack the genes encoding proline peptidases PepI, PepR or PepL. This agrees with the observation from gene deletion experiments in strains harboring those peptidase genes that the physiological role of PepI, PepR or PepL is not essential for cell growth [39,45,46]. However, in *L. helveticus*, the growth rate in milk was slower for a PepI-deletion mutant as compared to the wild type [45]. Similarly, the activity of cell extract of *L. helveticus *and *L. rhamnosus *toward several proline dipeptides was significantly reduced in a PepR-deletion mutant [39,46]. Those observations suggest that PepI/PepR/PepL may contribute specifically to the proteolytic capacity on proline-containing peptides of *Lactobacillus *strains.

### Diversity of the proteolytic system in *L. lactis *strains

The distribution of proteolytic system components in various *L. lactis *strains was studied by comparative genome hybridization (CGH) analysis. PanGenome arrays were made based on ORFs found in four sequenced *L. lactis *strains, and subsequently used to determine the presence or absence of orthologs in 39 *L. lactis *strains [14]. Table 1 summarizes only the proteolytic system genes with variable absence/presence patterns in the 39 *L. lactis *strains. All other components described in Figure 1 but not shown in Table 1, such as PepC, PepN, PepM, PepA, PepD, PepV, PepT, PepP, PepQ, DtpT and most members of the Dpp system, are present in almost all strains. PepE/PepG and PepI/PepR/PepL family members are absent in all *L. lactis *strains. Those genes are excluded from the table, as well as all genes of strains P7304 and P7266 (see explanation in Table legend). Some plant-derived *L. lactis *strains such as KF24, NIZOB2244W, LMG9446 and KW10 have the largest set of proteolytic system genes.

**Table 1:** Distribution of variable peptidases and peptide transporters in *Lactococcus lactis *strains by CGH analysis (genes which are either present or absent in all query and reference strains are excluded)

Strains ^a^	ssp	origin	PrtP	PrtM	Pcp	PepF1	OppA1	PepO2	PepF2	OppA2	OppB2	OppC2	OppD2	OppF2	DppB	PepX2 (YmgC)
KF24	lactis	plant	**1**	**1**	**1**	**1**	**1**	**1**	**1**	**1**	**1**	**1**	**1**	**1**	**1**	**1**

NIZOB2244W	lactis	plant	**1**	**1**	**1**	**1**	**1**	**1**	**1**	**1**	**1**	**1**	**1**	**1**	**1**	**1**

LMG9446	lactis	plant	**1**	**1**	**1**	**1**	**1**	**1**	**1**	**1**	**1**	**1**	**1**	**1**	**1**	**1**

KF196	lactis	plant	0?	0?	0?	**1**	**1**	0?	0?	0?	0?	0?	0?	0?	**1**	0?

KF201	lactis	plant	0?	0?	0?	**1**	**1**	0?	0?	0?	0?	0?	0?	0?	**1**	0?

N42	lactis	plant	**1**	**1**	0	**1**	**1**	0	0	0	0	0	0	0	**1**	**1**

KF134	lactis	plant	0	0	0	**1**	**1**	0	0	0	0	0	0	0	**1**	0

E34	lactis	plant	0	0	0	**1**	**1**	0	0	0	0	0	0	0	**1**	0

Li-1	lactis	plant	0	0	0	**1**	**1**	0	0	0	0	0	0	0	**1**	0

M20	lactis	plant	0	0	0	**1**	0	0	0	0	0	0	0	0	**1**	0

K231	lactis	plant	0	0	0	**1**	**1**	0	0	0	0	0	0	0	**1**	0

LMG9449	lactis	plant	0	0	0	**1**	**1**	0	0	0	0	0	0	0	0	**1**

KF146	lactis	plant	0	0	0	**1**	**1**	0	0	0	0	0	0	0	0	0

KF147	lactis	plant	0	0	0	**1**	**1**	0	0	0	0	0	0	0	0	0

K337	lactis	plant	0	0	0	**1**	0	0	0	0	0	0	0	0	0	0

KF67	lactis	plant	0	0	0	**1**	**1**	0	0	0	0	0	0	0	0	0

KF7	lactis	plant	0	0	0	**1**	**1**	0	0	0	0	0	0	0	0	0

LMG8520	lactis	plant	0	0	0	**1**	**1**	0	0	0	0	0	0	0	0	0

KF282	lactis	plant	0	0	0	0	0	0	0	0	0	0	0	0	**1**	0

LMG8526	lactis	plant	0	0	0	0	0	0	0	0	0	0	0	0	0	0

UC317	lactis	dairy	**1**	**1**	0?	**1**	**1**	0?	0?	0?	0?	0?	0?	0?	**1**	**1**

ML8	lactis	dairy	**1**	**1**	0	**1**	**1**	0	0	0	0	0	0	0	**1**	**1**

LMG14418	lactis	dairy	0	0	0	**1**	**1**	0	0	0	0	0	0	0	**1**	**1**

IL1403	lactis	dairy	0	0	0	**1**	**1**	0	0	0	0	0	0	0	**1**	**1**

DRA4	lactis	dairy	0	0	0?	**1**	**1**	0	0	0	0	0	0	0	**1**	**1**

NCDO895	lactis	dairy	0	0?	0?	**1**	**1**	0	0?	0?	0?	0	0	0	**1**	**1**

ATCC19435T	lactis	dairy	**1**	**1**	**1**	**1**	**1**	**1**	**1**	**1**	**1**	**1**	**1**	**1**	**1**	**1**

KW10	cremoris	plant	**1**	**1**	**1**	**1**	**1**	**1**	**1**	**1**	**1**	**1**	**1**	**1**	**1**	**1**

N41	cremoris	plant	**1**	**1**	**1**	**1**	**1**	**1**	**1**	**1**	**1**	**1**	**1**	**1**	**1**	0

NCDO763	cremoris	dairy	**1**	**1**	**1**	**1**	**1**	**1**	**1**	**1**	**1**	**1**	**1**	**1**	**1**	0

SK11	cremoris	dairy	**1**	**1**	**1**	**1**	**1**	**1**	**1**	**1**	**1**	**1**	**1**	**1**	**1**	0

AM2	cremoris	dairy	**1**	**1**	**1**	**1**	**1**	**1**	**1**	**1**	**1**	**1**	**1**	**1**	**1**	0

FG2	cremoris	dairy	**1**	**1**	**1**	**1**	**1**	**1**	**1**	**1**	**1**	**1**	**1**	**1**	**1**	0

LMG6897T	cremoris	dairy	**1**	**1**	0?	0	**1**	**1**	**1**	**1**	**1**	**1**	**1**	**1**	**1**	0

V4	cremoris	dairy	0?	0?	0?	0	**1**	**1**	**1**	**1**	**1**	**1**	**1**	**1**	**1**	0

HP	cremoris	dairy	0	0	**1**	**1**	**1**	**1**	**1**	**1**	**1**	**1**	**1**	**1**	**1**	0

MG1363	cremoris	dairy	0	0	**1**	0	**1**	**1**	**1**	**1**	**1**	**1**	**1**	**1**	**1**	0

a. Two *Lactococcus *strains P7304 and P7266 are not shown in this table since they are grouped separately from other strains phylogenetically [53] and present a distinct pattern of presence and absence of proteolytic genes. For instance, PepC, PepA, PepO, PepF, PepV, PepX, and PepP appear to be absent in these two strains, but this could also be the result of poor hybridization due to lower sequence homology.

? The score of the signal is not significant enough to conclude the presence or absence of a gene. Most of the assignments of absence/presence are assumed based on coexistence patterns of genes, e.g. PrtP and PrtM, or OppABCDF are usually encoded in the same operons, thus should be all present or all absent in the genomes. In these cases, a stricter cut-off value for deciding the presence of a gene (5.6-5.7 instead of 5.5) is used.

Variations are found for proteinase PrtP and its maturation protein PrtM, for peptidases Pcp, PepO2, PepF2 and PepX2, and for genes from peptide transport systems Opp and Dpp (Table 1). Most of these genes are known to be present on plasmids [27]: in strain SK11 the *prtP*, *prtM *and *pcp *genes are located on one large plasmid, while the *pepO2*, *pepF2 *and *oppABCDF2 *are co-localized on a different plasmid. The co-presence or co-absence of these genes in other *L. lactis *strains (Table 1), is largely consistent with their coupling in SK11, and suggests that variability is mainly due to the presence or absence of the plasmids. Cell-wall bound proteinase PrtP together with PrtM are mainly present in *L. lactis *subsp. *cremoris*, although several *L. lactis *subsp. *lactis *strains also harbor these genes (including dairy strains e.g. UC317, ML8, and ATCC19435T).

PepX2 is a PepX homolog of *L. lactis *subsp. *lactis *IL1403. It is mainly found in *L. lactis *subsp. *lactis *strains from dairy origin. This putative *pepX2 *gene was originally annotated as a hypothetical protein named YmgC. It contains both a C-terminal domain of X-prolyl dipeptidyl aminopeptidase and a Peptidase_S15 catalytic domain which are usually found in PepX, whereas the PepX N-terminal domain is missing in PepX2. No experimental evidence for the enzyme activity of PepX2 is known. The family tree of PepX shows that this putative *pepX2 *gene is not clustered in the same orthologous group as its paralogous gene from *L. lactis *subsp. *lactis *IL1403 [Additional File 5]. The only members of the PepX2 (YmgC) group in sequenced LAB genomes are from *L. lactis *subsp. *lactis *IL1403 and *Pediococcus*. Their best BLAST hits against the non-redundant protein database are from *Listeria monocytogenes*, suggesting a HGT event [See Additional File 5].

## Discussion

In this study, we performed a systematic genome-wide analysis of all the proteins involved in proteolysis, including cell-wall bound proteinase, peptide transporters, and peptidases, from twenty-two fully sequenced LAB genomes, including *Lactobacillus, Lactococcus, Streptococcus, Pediococcus, Oenococcus*, and *Leuconostoc *strains. The comparative genomics analysis was shown to distinguish various subgroups within a protein superfamily, allowing a highly improved annotation of genes and clarification caused by inconsistent annotation.

This information on the distribution of the proteolytic system genes can be used to predict the proteolytic potential of various LAB strains. For instance, *L. bulgaricus *and *L. helveticus *have a very extensive set of proteolytic enzymes, which is consistent with previous knowledge that *L. bulgaricus *serves as the proteolytic organism in yoghurt rather than *S. thermophilus *[47]. *L. helveticus *is a proteolytic cheese adjunct culture that has been used to degrade bitter peptides in cheese [48]. Interestingly, *L. bulgaricus *encodes the Dpp system with preference for uptake of hydrophobic di/tripeptides, complementing *S. thermophilus *which encodes the general di/tripeptide transporter DtpT in its genome, suggesting that more peptides can be utilized by both bacteria when grown together. LAB species of plant origin, such as *L. plantarum, O. oeni*, and *Leuconostoc mesenteroides*, encode less proteolytic enzymes in their genomes, which agrees with their ecological niche that is fiber-rich but contains less proteins.

Several examples have been provided for the division of large superfamilies into subfamilies. Clear separation of major subgroups can be observed from the family trees. By including the experimentally characterized genes, different substrate specificities can be assigned to various subfamilies. The PepP/PepQ/PepM and PepI/PepR/PepL superfamilies include subfamilies with distinct substrate specificities. The general dipeptidase superfamily PepD consists of several distinct orthologous groups of which the substrate specificities are still unknown. In most cases, the prediction of orthologous groups and the evolutionary events leading to the variation of substrate specificities are straight-forward using the phylogenetic analysis. However, some orphan genes are present as intermediate groups between the subfamilies with unknown functions and some of them may originate from HGT events.

Peptidases PepI/R/L and the esterase EstA, which is also involved in flavor-formation by LAB, belong to the same α/β hydrolase superfamily. We performed a comparative analysis of 3D structures of representative proteins from each subfamily in order to identify the core regions of the enzymes and to improve the multiple sequence alignment of the superfamily. Orthologs could then be identified more clearly in the protein family tree as constructed on basis of the curated MSA of the core regions. The classic catalytic triad Ser-His-Asp of the α/β hydrolase family is conserved in most of the members of the PepI/R/L and EstA superfamily. However, in the PepR subfamily of LAB (Figure 5), the catalytic Asp residues are substituted by Glu residues. Aspartate and glutamate residues are chemically equivalent and differ only in length of the side chain. The substitution of Asp by Glu has been observed in prokaryotic subtilases [49], as well as in an acetylcholinesterase of *Torpedo californica *and a lipase of *Geotrichum candidum *[50,51]. Moreover, two additional peptidases from *L. plantarum *and *L. casei *(LPL_28379307 and LCA_116494294) which are not grouped into the PepR subfamily (Figure 5) also have glutamate catalytic residues instead of aspartate residues. It suggests that the substitution of Asp to Glu may have happened in the common ancestor of these two proteins and the PepR family. Since the glutamate residue at the catalytic triad is only found to be conserved in the PepR subfamily, it can be used as an extra indication for determining whether a peptidase with unclear function belongs to the PepR subfamily.

One of the applications of our comparative analysis is to explore the diversity of proteolytic system genes in various strains of *L. lactis *by combining the results from comparative genomics analysis and the hybridization data from pangenome CGH analysis. Distinct patterns were found in the presence and absence of proteolytic enzymes in the two *L. lactis *subspecies, *i.e*. subsp. *lactis *and subsp. *cremoris*, confirming the proteolytic diversity between the subspecies, and now providing a genetic basis for this diversity. Several strains show corresponding distributions of some proteolytic genes in their genomes, presumably resulting from the presence or absence of plasmids encoding proteolytic system components.

## Conclusions

We performed a genome-wide comparative study on the proteolytic system of LAB, and demonstrated that the functional annotation of proteolytic system genes can be improved by combining phylogeny, synteny and literature. Examples of the PepP/PepQ/PepM family, the PepD family and the PepI/PepR/PepL family elucidated that protein subfamilies with distinct substrate specificities can be identified. In the case of the PepI/PepR/PepL family, protein 3D-structure alignment allowed us to more clearly distinguish the peptidase subfamilies and an esterase family EstA. Moreover, the complete distribution of proteolytic system components in various sequenced LAB strains was obtained.

The diversity of proteolytic system genes from 39 *Lactococcus *strains was explored using CGH analysis. Several components including proteinase, oligopeptide transport system and peptidases were shown to be distributed unevenly among the *Lactococcus *strains. The presence or absence of those proteolytic system components are probably the result of the presence or absence of plasmids that encode them.

Knowledge of the variations in proteolytic system components may allow the prediction of proteolytic and flavor-forming potential of bacterial strains, and could direct future experimental tests into the phenotypes of various LAB. Ultimately, this knowledge could be used to improve the sensory characteristics of dairy and other fermented food products by supporting the strain selection process.

## List of abbreviations used

LAB: Lactic Acid Bacteria; HMMs: Hidden Markov Models; MSA: Multiple Sequence Alignments; CGH: Comparative Genome Hybridization; HGT: Horizontal Gene Transfer.

## Authors' contributions

ML conceived and designed the study, performed the analyses, drafted and revised the manuscript; JRB carried out the pangenome CGH analysis and the diversity analysis of *Lactococcus lactis*; BR carried out the protein 3D structure alignment; AN coordinated the study and helped revising the manuscript; RJS conceived, designed and coordinated the study, helped drafting and revised the manuscript. All authors read and approved the final manuscript.

## Supplementary Material

Additional file 1 **Multiple sequence alignment of core regions of proteins from both the PepI/R/L and EstA families**. A manually curated multiple sequence alignment of the concatenated sequences of the four core regions of the PepI/PepR/PepL and EstA superfamily members identified by the protein structure superposition. On basis of this MSA, a family tree was constructed, and is shown in Figure 5. Click here for file

Additional file 2 **Proteolytic components in LAB**. The file contains a table with GI codes of all the genes listed in Figure 1. Click here for file

Additional file 3 **Superfamily tree of PepF members**. The bootstrapped (n = 1000) neighbor-joining tree for PepF members in LAB Click here for file

Additional file 4 **Comparison of important residues of the conserved core regions and the cap region**. The file contains a table describing the four structurally conserved regions and the cap regions. The identified residues within those regions, which are functionally important and/or conserved in PepI/R/L or EstA families, are highlighted Click here for file

Additional file 5 **PepX superfamily tree**. The file contains a bootstrapped (n = 1000) NJ tree for PepX family homologs of LAB Click here for file
