# Supplementary material for: The proteolytic system of lactic acid bacteria revisited: a genomic comparison
Source: BMC Genomics. 2010 Jan 15;11:36. doi: 10.1186/1471-2164-11-36 (PMC2827410; doi:10.1186/1471-2164-11-36)
Supplement: Additional file 1 — Multiple sequence alignment of core regions of proteins from both the PepI/R/L and EstA families. A manually curated multiple sequence alignment of the concatenated sequences of the four core regions of the PepI/PepR/PepL and EstA superfamily members identified by the protein structure superposition. On basis of this MSA, a family tree was constructed, and is shown in Figure 5. [file 1471-2164-11-36-S1.DOC]

**Additional File 3.** Multiple Sequence Alignment of the core regions of proline peptidases and esterase A. Four conserved sections of the core region and the cap region are indicated by red dashed lines. Catalytic residues are highlighted in yellow. The number of residues in the cap region and between sections is shown. Residue numbering corresponds to the concatenated sequences starting at section I.

|-------------------------------------SECTION I-------------

5 10 15 20 25 30 35 40 45 50 55 60

| | | | | | | | | | | |

MLO_13476474 -14-VESGMLDVGD-GHQVYWERSGTK--------------GAKPAVFLHGGPGGTISPK--HR

YPI_108812503 -13-YDSGLLDTGD-GHQIYWELCGNP--------------KGKPAIFIHGGPGGGIAPY--HR

1WM1 -13-YDSGWLDTGD-GHRIYWELSGNP--------------NGKPAVFIHGGPGGGISPH--HR

1AZW -10-YQQGSLKVDD-RHTLYFEQCGNP--------------HGKPVVMLHGGPGGGCNDK--MR

XFB_170730035 -10-FEHGMLCVDD-SHRLYYEQCGNP--------------HGKPVVILHGGPGGGCNDK--MR

MX_108757707 -17-YRTGRLRVSG-GHEVYFEESGNP--------------EGKPVLFVHGGPGGGTDPR--QR

PA_15600273 -10-YARHELAVDE-PHVLYADESGSP--------------DGLPVVFVHGGPGSGCDAL--SR

PF_70733915 -10-YARHDLAVDE-PHVLYVDESGSP--------------EGLPVVFIHGGPGAGCDGA--SR

BCR_42783975 ------MFVTVEKD-VHIFVQDVNPGP--------------SSKTVFFFHSWPLNHQMYQ--YQ

BS_16078154 ------MGHYIKTE--EHVTLFVEDIG--------------HGRPIIFLHGWPLNHKMFE--YQ

CAC_15895946 ------MGYYIKVE-SDVKLYIEDVNP-------------RANKTILFLHGWPGSHKLFE--YQ

OOE_116490346 -01-KNGTHILTLT-SGFHLWSHTENS-------------GKKSKIIAVHGGPGETHESF---E

LGA_116630389 -11-ANESKIVTLK-NGYHVWTRKEGH--------------GPIKILLLHGGPGMDHEYL-EPF

OOE_116490640 -02-HQTKILHLK-NGYDIWSASFGNP-------------DAPIKLLTLHGGPGGTSKEFWPWA

OOE_116491087 -02-HKTKILHLK-NGYDLWGASFGDP-------------NSPIKILALHGGPGDDADEFIVWA

LPL_28379370 -03-VTRILTLSN-GYHLWSHTSNL---------------GGRTKLLCLHGGPGDTHEVF---E

LDE_pepL -02-QTRIVTLDN-GYHLFTRKVN-----------------EGVKLLCVHGGPGDNHEDF---D

LAC_58338197 -02-QTRLVTLDN-GYHLFTRKVNE----------------GPIKLLCLHGGPGGTHETF---D

LCA_116494294 -07-GTQFITLSS-GYHLWTQTQGK----------------GDIHLMTLHGGPGGTNEVF---E

LRF_148531889 -03-GTKIITLDN-GYHLWTNTQGE----------------GDIHLLALHGGPGGNHEYW-EDA

LBU_116514722 -03-GTTILTLDN-GYHLWTNTQGT----------------GDIHLLCLHGGPGGNHEYW-EDT

LAC_58337916 -03-GTKIITLDN-GYHLWTNTQGE----------------GDIHLLALHGGPGGNHEYW-EDT

LHE_pepR -03-GTKIITLDN-GYHLWTNTQGE----------------GDIHLLALHGGPGGNHEYW-EDT

LJO_42518140 -03-GTKIITLDN-GYHLWTNTQGE----------------GDIHLLALHGGPGGNHEYW-EDA

LGA_116628733 -03-GTKIITLDN-GYHLWTNTQGE----------------GDIHLLALHGGPGGNHEYW-EDA

LSK_81427743 -03-GTTILTLDN-GYHLWTNTQGT----------------GDIHLLCLHGGPGGNHEYW-ENF

PPE_116492182 -03-GTTILTLDN-GYHLWSHTDGD---------------DDSIHLLALHGGPGGNHEYW-ENF

LBE_116334352 -03-GTTIITLDN-GYHLWTNTQGT----------------GDIHLLALHGGPGGNHEYW-EDT

LPL_28377695 -03-GTTIITLDN-GYHLWTNTQGK----------------GDIQLLCLHGGPGGNHEYW-ENF

LCA_116495454 -03-GTTILTLDN-GYHLWTNTQGE----------------GDIHLLCLHGGPGGTHEYW-ENF

1MTZ -04-CIENYAKVNG--IYIYYKLCKAP-------------EEKAKLMTMHGGPGMSHDYL--LS

CBG_170761364 -02-ITEGYMPYLE--YKTYYRIVGEC------------TGNKKPLVLLHGGPGSTHNYF--EV

LPL_28377046 -02-VQEGYMPFNG--YQTYYRIVGDR------------QSNKTPLVLLHGGPGSTHNYF--EG

LBE_116334786 -02-IQEEYMPFRA--YQTYYRVVGDL------------RSPLTPLLLLHGGPGSTHNYF--EA

LDE_pepI -03-ITEKYLPFGN--WQTYCRIVGEA-------------TDRAPLLLLHGGPGSSHNYF--EV

LBU_116514711 -03-ITEKYLPFGN--WQTYCRIVGEA-------------TDRAPLLLLHGGPGSSHNYF--EV

LAC_58336439 -02-IIEGKMPFMG--YETHYRIVGRR-------------SEKSPLVLLHGGPGSTHNYF--EV

LHE_161506712 -02-IIEGKMPFMG--YETYYRIVGER-------------SEKPPLVLLHGGPGSSHNYF--EV

MAI_118464592 --------MVAVPG--GQVWFQRTGG--------------GAGLPLLVIHGGPGLPHDYL--RS

RMTG03682 -13-QVEGTIAVPG--GRVWFQRIGG--------------GPGRPLLVVHGGPGLPHNYL--AP

RSTS00294 -03-TYLYGEMFSENILTSYRIYGDR-------------KSLLTPLIILHGGPSGGFDYL--LN

REM_190894576 -15-ATNEAYLPFRDYRTWYRITGSL-------------ESGKLPLVVAHGGPGCTHDYV--DS

XCP_66767623 -01-QCTEGFVEFRGYRTWYRITGDL-------------HSDACPLIVLHGGPGCTHDYV--DS

LCA_est -02-LLHLDFKSTALRHSTSLYAIIPEVT------ENFSRDQYQVLYLLHGMGDDYTKWVRRTN

LLC_estA -02-VINIEYYSEVLGMNRKVNVIYPESSKV----EDFSNTEIPVLYLLHGMSGNENSWMIRSG

LLA_estA -02-VINIEYYSEVLGMNRKVNVIYPESSKV----EDFTQTDIPVLYLLHGMSGNENSWIIRSG

OOE_116491533 -02-FLQINYHSHVLGKATMMNVILPELDTNN---NNNKRRDIPVLYLLHGMGDDLFSWQRETN

OOE_116490775 -02-FLEVNYYSRVLGMNRVMNVLLPEESDHNPNWTNDSLKDLPVLYLLHGMSGNHFDWQRKSD

LME_116618577 -02-FLEVNYYSKVLGMDRVMNVILPELSDHNPTWTTETLKDIPVLYLLHGMSGDHAIWQRRTS

2UZ0 -06-VMKIEYYSQVLDMEWGVNVLYPDANRV----EEPECEDIPVLYLLHGMSGNHNSWLKRTN

STU_55821195 -02-FFQIEYSSVVLGQYRQVDVIYPDRDQIA---ETESDTDIPVLYLLHGMGGNHNSWAFRTN

--------------------| |--SECTION II-| |----SECTION III----

65 70 75 80 85 90 95 100 105 110 115 120

| | | | | | | | | | | |

MLO_13476474 RL--FDP-KLYDVILFDQRG-17-LVADI-ERLREMAG-03-WLVFGGSWGSTLALAYAE--

YPI_108812503 QL--FNP-AKYNVMLFDQRG-17-LVEDI-ERLRKMAG-03-WLVFGGSWGSTLALAYGE--

1WM1 QL--FDP-ERYKVLLFDQRG-17-LVADI-ERLREMAG-03-WLVFGGSWGSTLALAYAQ--

1AZW RF--HDP-AKYRIVLFDQRG-17-LVADI-ERLRTHLG-03-WQVFGGSWGSTLALAYAQ--

XFB_170730035 RF--HDP-DKYRIVLFDQRG-17-LVADI-EKLRVALG-03-WQVFGGSWGSTLALAYAQ--

MX_108757707 RF--FDP-TAYRIILFDQRG-17-LVADM-ERLREFLD-03-WQLFGGSWGSTLSLAYAQ--

PA_15600273 RF--FDP-NLYRIVTFDQRG-17-LVADM-ERLREHLG-03-WVLFGGSWGSTLSLAYAQ--

PF_70733915 CY--FDP-NLYRIVTFDQRG-17-LVADL-ERIRQHLG-03-WVLFGGSWGSTLALAYAQ--

BCR_42783975 LN--VLPQHGFRCIAMDIRG-15-LADDI-AIVLEALQ-03-ATLVGFSVGGALSIRYMS--

BS_16078154 MN--ELPKRGFRFIGVDLRG-15-MADDV-KAVIYTLQ-03-AILAGFSMGGAIAIRYMA--

CAC_15895946 FD--QLPKRGYRCIGIDQRG-15-LSDDV-RCVVETLS-03-FTLAGHSTGGAIAIRYMA--

OOE_116490346 TL--PLGVPNAEITSYDQLG-23-FVNEL-EEVRRQLE-03-FILLGYSWGAMIALEYAL--

LGA_116630389 SDY-IKIHPEIEIIYYDQLG-17-FIEEI-EEVRKAWN-03-FYLYGQSFGGLFALEYAA-S

OOE_116490640 ENFQKYVGMDVQVFTYDQLG-23-YLDEL-EEVRSLMG-03-FYLLGHSWGGVLTYEYSL--

OOE_116491087 DQLKKFARIDAEVFVYEQLG-23-YINEV-EEVRQLFD-03-FYLAGHSWGGILAYEYMLRS

LPL_28379370 RFGPELADLDIEVTMYDQLG-23-YLSEV-DEVRQQLG-03-CYLAGHSWGGMLAMTYAA--

LDE_pepL NFKAGLAGKGVEVYSYDQLG-22-FVDEL-EEVRQKLG-03-FYLLGHSWGGLLAQEYAV--

LAC_58338197 NFKDGLKGQGVEVYSYDQLG-22-YVDEV-EEVRQKLG-03-FYLLGHSWGGLLAQEYAY--

LCA_116494294 NFAERLAPYGVRVTRYDQLG-23-YLSEV-EQVRQQLG-03-FYLLGQSWGGVLAIEYAL--

LRF_148531889 AEQLKKQGLNVQVTMYDQLG-23-FLDEV-DEVREKLG-03-FYLIGQSWGGLLVQEYAV--

LBU_116514722 AEQLKKQGLNVQVHMYDQLG-23-FLDEV-EEVRQKLG-03-FYLIGQSWGGLLVQEYAV--

LAC_58337916 AEQLKKQGLDVQVTMYDQLG-23-FLDEV-DEVREKLG-03-IYLIGQSWGGLLVQEYAV--

LHE_pepR AEQLKKQGLNVQVTMYDQLG-23-FLDEV-DEVREKLG-03-FYLIGQSWGGLLVQEYAV--

LJO_42518140 AEQLKKQGLNVQVTMYDQLG-23-FLDEV-DEVREKLG-03-FYLIGQSWGGLLVQEYAV--

LGA_116628733 AEQLKKQGLNVQVTMYDQLG-23-FLDEV-DEVREKLG-03-FYLIGQSWGGLLVQEYAV--

LSK_81427743 GKELAD--LGVQVHMYDQLG-22-FLDEV-EEVRQKLG-03-FYLIGQSWGGALVQMYAA--

PPE_116492182 HDELLKQGLNVQVHYYDQLG-23-FLDEV-EEVRQKLG-03-FYLIGQSWGGALTMMYAL--

LBE_116334352 AKQLAAQGLNVQVHMYDQLG-23-YLDEV-EEVRQKLG-03-FYLIGQSWGGALVQMYAA--

LPL_28377695 GEELAD--LGVQVSMYDQLG-23-FLDEV-EEVRQKLG-03-FYLIGQSWGGALTMMYAL--

LCA_116495454 GEELAD--LGVQVHMYDQLG-23-FLGEV-EEVRQKLG-03-FYLIGQSWGGALTQLYAL--

1MTZ LRD-MTK-EGITVLFYDQFG-16-GVEEA-EALRSKLF-04-VFLMGSSYGGALALAYAV--

CBG_170761364 LDK-VAE-DGRAVIMYDQLG-17-WIEEL-VQLRKHLG-03-IHLLGQSWGGMQAIQYAC--

LPL_28377046 FDD-LAAQTGRPIVMYDQLG-17-WVAEL-RALRTYLD-03-IHLLGQSWGGMLAIIYGC--

LBE_116334786 FDQ-LAMATGRPIVMYDQLG-17-WVAEL-RALRAYLK-03-VHLLGQSWGGMLALIYLC--

LDE_pepI LDQ-VAEKSGRQVIMYDQLG-19-WVKEL-ENVREQLG-03-IHLLGQSWGGMLALIYLC--

LBU_116514711 LDQ-VAEKSGRQVIMYDQLG-19-WVKEL-ENVREQLG-03-IHLLGQSWGGMLALIYLC--

LAC_58336439 LDK-LAKIDDRRIIMYDQLG-18-WVKEL-KTLREHLA-03-IHLLGQSWGGMLAIIYMC--

LHE_161506712 LDE-LAQKDGRRIIMYDQLG-18-WVKEL-EALREHLA-03-MHLLGQSWGGMLAIIYMC--

MAI_118464592 LRR-LATD--REVIFWDQLG-17-SVAEV-DAVVRALR-03-FHLFGNSWGGMLAQQYVL--

RMTG03682 LRR-LSDE--REVIFWDQLG-17-SVAEM-ATVAEALA-03-FHIFSHSWGGMLAQQYVL--

RSTS00294 YRR-LADDG-RMVIFYDQYG-19-YLRQL-TQLIHHLG-05-YSILGHSWGGMLAAEHAC--

REM_190894576 FKD-IAALDGRPVIHYDQLG-19-FLEEL-DALLTHIG-04-YAFLGQSWGGMLGAEHAV--

XCP_66767623 FTD-LAGSG-RAVIHYDQLG-19-FLDEL-QALISHLG-03-YALLGQSWGGMLAAEHAV--

LCA_est IEQ-YVVTISWSSLSAGFAN-11-YWTFLTEELPSITN-11-HFVAGMSMGGFGAFKWA--L

LLC_estA IER-LIRHTNLAIVMPSTDL-11-YFDAIALELPKVIH-12-NFIAGLSMGGYGAYRLA--L

LLA_estA IER-LIRHTNLAIVMPSTDL-11-YFDAIAHELPKVIN-12-NFIAGLSMGGYGAYRLA--L

OOE_116491533 IER-LLMKNNLAVVMPDTGL-11-YFDALTAELFQKVA-12-HFVAGLSMGGYGAFKLA--M

OOE_116490775 IER-LLRQTKLAVIMPAADL-11-YFDAISQELPRKVA-12-HFVAGMSMGGYGAFKLA--F

LME_116618577 IER-LVRQTPVAIVMPSTDL-11-YFDALARELPEKVA-12-NFVAGLSMGGYGAFKLA--L

2UZ0 VER-LLRGTNLIVVMPNTSN-11-YYTALAEELPQVLK-12-TFIAGLSMGGYGCFKLA--L

STU_55821195 IQR-LLRKTNLIVIMPNSEN-11-YYDAIAKELPQVMQ-12-TFIAGLSMGGYGSFKIA--L

----------------|CAP |------------------------SECTION IV------

125 130 135 140 145 150 155 160 165 170 175 180

| | | | | | | | | | | |

MLO_13476474 THP-DRVSELVVRGIY-107-QLLRDA-WKLK----DIPGTIVHGRYDMPCPARYAWALHKA

YPI_108812503 THP-ERVSEMVLRGIF-107-QLLDNV-TRIR----HIPAVIIHGRYDMACQLQNAWDLAQA

1WM1 THP-ERVSEMVLRGIF-095-QLLRNV-PLIR----HIPAVIVHGRYDMACQVQNAWDLAKA

1AZW THP-QQVTELVLRGIF-097-QLLRDA-HRIA----DIPGVIVHGRYDVVCPLQSAWDLHKA

XFB_170730035 THP-EQTTELVLRGIF-108-QLLRDA-QRIA----NIPGVIVHGRYDVVCPLQNAWDLHKA

MX_108757707 THP-ERVSELVLRGIF-108-QLLDDV-HRIR----KIPAVIVQGRYDVVCPPESAWALHKA

PA_15600273 THP-ERVHALILRGIF-107-QLLEDM-HRIA----HLPGVIVHGRYDAICPLDNAWALHQA

PF_70733915 THP-ERVLGLIVRGIF-107-QLIRDM-GKIA----HLPGVIVHGRYDVICPLDNAWELHQA

BCR_42783975 RYNGQRISKLVLIDAV-076-DVTKDL-SKI-----NVPTKIFHGIHDQLIPYKSAELTQKR

BS_16078154 RHEGADVDKLILLSAA-075-DLRKEL-AAI-----KVPTLILHGRKDRIAPFDFAKELKRG

CAC_15895946 RHKQYGVNKLALFAAA-097-ELFSDL-KKI-----TVPTLILHGIHDKVCLFPLAEAQKRG

OOE_116490346 KYP-GNLDKLVIVGMS-080-NVSDKL-YRI-----KTPTLILVGDQDMI-SPKKARVMADK

LGA_116630389 KYG-KHVKALIDSNMV-081-TIRDRL-SKI-----TMPTLVLGGKYDSM-NPDDIKALADR

OOE_116490640 AHP-EHLKGSIVYSMT-080-NIRPRL-HNI-----KIPMLLTTGEKDTM-PVWSMKETAKT

OOE_116491087 EYR-QHLKAGIVFSMN-080-NVSSRL-PEF-----DIPVLETVGEFDTM-SVESAKRSARK

LPL_28379370 DHQ-DQLDGLIIISMI-080-DFSDTL-ATI-----QVPTLLMFADHETM-PLATAERMQQR

LDE_pepL RYG-QHLKAVVIESMI-080-DFRDKL-AQI-----TLPTLLTVGEFDTM-PLDAVRRMHHS

LAC_58338197 KYG-KHLKGLVLMSMI-079-DFREKL-ASL-----KMPTLLTFGEFDTM-PLSAARRMHQT

LCA_116494294 KYP-EHLNGVILSSMI-080-DRRADI-HRI-----AVPTYLTFGGHETM-PLAAAERMAKT

LRF_148531889 KYG-QHLKGAIISSMV-080-HFRDQL-KNI-----KVPTLITFGEHETM-PIETAKTMNSL

LBU_116514722 KYG-DHLKGAIISSMV-080-HFRSEL-PKI-----KVPTLLTFGEVETM-PLETARTMQKL

LAC_58337916 KYG-QHLKGAIISSMV-080-HFRDQL-HKI-----NVPTLLTFGENETM-PISTAKIMQKE

LHE_pepR KYG-QHLKGAIISSMV-080-HFRDQL-KNI-----KVPTLLTFGENETM-PISTAKIMQKE

LJO_42518140 KYG-QHLKGAIISSMV-080-HFRDQL-KNI-----KVPTLITFGENETM-PISTAKIMQKE

LGA_116628733 KYG-KHLKGAIISSMV-080-HFRDQL-KNI-----KVPTLITFGENETM-PISTAKIMQKE

LSK_81427743 KYG-QHLKGAIISSMV-080-HFRDQL-HKI-----TVPTLITFGEHETM-PIATAKIMAEK

PPE_116492182 KYG-QHLKGAIISSMV-080-DVRDQI-KNI-----NVPTLLTFGEHETM-PLDSARRMAEV

LBE_116334352 KYG-QHLKGAIISSMV-080-NFRDHL-KDI-----QVPTLLTFGEHETM-PLATGQKMAEL

LPL_28377695 KYG-QHLKGAIISSMV-080-DIRDQI-HNI-----KVPTLLTFGEHETM-PLASARRMARD

LCA_116495454 KYG-QHLKGAIISSMV-080-DISDQI-HNI-----KVPTLLTFGEHETM-PLKSAQRMAEV

1MTZ KYQ-DHLKGLIVSGGL-092-DITDKI-SAI-----KIPTLITVGEYDEV-TPNVARVIHEK

CBG_170761364 EYKPEGIKSYILSSTL-092-DFMKEI-EDI-----KEPCLITSGLLDLC-SPLVAKTMYDK

LPL_28377046 DYRPQGIKSLILASTL-082-EYTDRL-QYL-----QMPTLVTSGTDDLC-TPLVAKTMVDQ

LBE_116334786 DDQPRGIQSVILASTL-082-DYTAKL-AQL-----PYPTLVTSGVNDLC-TPLVAKTMVDQ

LDE_pepI DYQPEGVKSLILSSTL-080-EYTDRL-KDL-----HLPALITSGTDDLC-TPLVAKSMYDN

LBU_116514711 DYQPKGVKSLILSSTL-081-EYTDRL-KDL-----HLPALITSGTDDLC-TPLVAKSMYDH

LAC_58336439 DYHPEGIQSLILSSTL-081-EYTDQL-SKI-----KVPTLITSGTDDLC-TPYVAKTMHDH

LHE_161506712 DYHPEGIQSLILSSTL-081-EYTDKL-SKI-----KVPTLITSGTDDLC-TPYVAKTMQDQ

MAI_118464592 DAAPAGAASLTISNSI-091-DVFDRL-GEI-----AVPTLVLAGRYDEC-VPEHMWEMHRR

RMTG03682 DKA-PDAVSLTIANST-091-DVVDRL-ADI-----AVPTLLVVGRFDEC-SPEHMREMQGR

RSTS00294 LQP-AGLRGTILASSP-093-DITPHL-CQI-----RCPVLVLRGENDQA-TERVVSPLLSH

REM_190894576 RRP-PGLKALVIANSP-093-TIEDRL-DRI-----GAPTLLISGKYDEA-TPLVVKPYLER

XCP_66767623 RRP-AGLRALVIANSP-093-SIIERL-HRI-----TAPTLVLSGKYDEA-TPETVEPYARL

LCA_est NKP-EMFEVAGSFSGV-106-DLSWLLTHQQDQ--ELPELYQYCGTEDP--ILQFNQKFADT

LLC_estA GT--DHFSYAASLSGV-032-EILALADRKNE---ERPKLYAWCGKQDF--LFPGNEYAIAE

LLA_estA GT--DYFSYAASLSGV-032-EILSLADRKQE---NKPKLYAWCGKQDF--LFPGNEYATAE

OOE_116491533 ST--DYFSYAASLSGA-034-DLFALAKKQSEGKAELPKLFAWIGLEDS--LYPANQFAIPT

OOE_116490775 SS--SYFSYAASLSGT-034-DIFELAKRQSNTGIELPKLYAWVGQQDF--FYGANEKAIPR

LME_116618577 GT--SQFSYAASLSGA-034-DILALAKTCH----KRPKLYAWIGEQDF--LKPINDVAIST

2UZ0 TT--NRFSHAASFSGA-034-SLESLAKKSD----KKTKLWAWCGEQDF--LYEANNLAVKN

STU_55821195 TT--NNFACAGSFSGA-034-KLVNLAKQHD----KKTKFFAWCGLEDF--LFDTQDQAVAD

----------------------------------------|

185 190 195 200 205 210 215 220

| | | | | | | |

MLO_13476474 W----PKADFHLIEGAGHAY-SEPGILD--RLIRATDKFA-02-

YPI_108812503 W----PEAELYIVEGAGHSF-DEPGILH--QLILATDKFA-01-

1WM1 W----PEAELHIVEGAGHSY-DEPGILH--QLMIATDRFA-02-

1AZW W----PKAQLQISPASGHSA-FEPENVD--ALVRATDGFA

XFB_170730035 W----PKASLKITPGAGHSA-FEPQNID--ALVCATDSFV

MX_108757707 W----PEAEFVMVSDAGHSA-NEPGNTS--ALVDATDRFR-02-

PA_15600273 W----PNSELQIIRDAGHTA-SEPGIVD--ALVRATNEIG-11-

PF_70733915 W----PNSELQVIRDAGHAA-SEPGITD--ALVRATDQMA-11-

BCR_42783975 I----KNSQLHPLTNSGHGSPIDQADE---LNEELIKFLH-01-

BS_16078154 I----KQSELVPFANSGHGAFYEEKEK---INSLIAQFSN-01-

CAC_15895946 I----KNSKLVRFEESGHGLFYDEKDR---LNSELMKFIE-01-

OOE_116490346 L----PNGKLEIIPDATHVSLRDNPEY---FFQQLNQFLT-01-

LGA_116630389 L----PNGTAHICPNGSHFSIFDDQED---YFNAITNFIT-05-

OOE_116490640 I----PGAELFVNKDGGHHHAVDHPVE---FYNNLAAFLK-06-

OOE_116491087 L----PRGRFALTENGGHSHAQDHPKE---FFEHLGSFIK-07-

LPL_28379370 M----PNAKLVVTPDSGHNHMVDNPAV---FFTYLRNYFS-05-

LDE_pepL L----KNSRMVVTPDGATATMLTTQMHFSLPYISSSVMLR-06-

LAC_58338197 L----SNSRLTLTPDGGHCHNTDNPKA---FFTSLTKFLH-10-

LCA_116494294 I----PDATLHVTPNAGHGQMLDNPTD---YFSHLGDWLV-11-

LRF_148531889 I----PNSQLVTTPDGGHHHMVDNPDV---YYKHLADFIR-09-

LBU_116514722 I----PNSRLVTTPDGGHHHMVDNPDV---YYKHLADFIR-10-

LAC_58337916 I----PNSRLVTTPDGGHHHMVDNPTV---YYKHLADFIR-11-

LHE_pepR I----PNSRLVTTPDGGHHHMVDNPDV---YYKHLADFIR-10-

LJO_42518140 I----PNSRLVTTPDGGHHHMVDNPDV---YYKHLADFIR-10-

LGA_116628733 I----PNSRLVTTPNGGHHHMVDNPDV---YYKHLADFIR-10-

LSK_81427743 I----PHSRLVTTPNGGHHHMIDNAPV---YFDHLKTFIK-09-

PPE_116492182 I----PNAKLVTTPNGGHHHMIDNAPV---YFKHLADYLR-09-

LBE_116334352 I----PHSRFVTTPEGGHHHMIDNAPV---YYDHLATFIR-09-

LPL_28377695 I----PNSRLVTTPNGGHHHMIDNAPV---YFDHLKQFIR-10-

LCA_116495454 I----PHARLVTTPDGGHHHMIDNAPV---YFKHLKQFIS-09-

1MTZ I----AGSELHVFRDCSHLTMWEDREG---YNKLLSDFIL-03-

CBG_170761364 I----PNSEWELFEFSRHMPFVEENEK---YIEVLNKWLN-03-

LPL_28377046 L----PHATWTLFPRSRHMAFIDENTA---YMTRLRHWLA-03-

LBE_116334786 L----PHAEWTLFPHSRHMAFIDEPAA---YQARLTQWLA-08-

LDE_pepI L----PNARWELFAGCGHMPFVQENAK---YQELLSDWLI-03-

LBU_116514711 L----PNARWELFAGCGHMPFVQENAK---YQELLSDWLI-03-

LAC_58336439 I----AGSQWKLFENCSHMSFVQKTDE---YIAMLKKWLD-03-

LHE_161506712 I----ASSKWRLFEGCGHMSFVEKTDE---YVALLQEWLD-04-

MAI_118464592 I----PGSRFELFESSAHMPFIEEPEK---FDAVMRDFLR-03-

RMTG03682 I----AGSRLEFFESSSHMPFIEEPAR---FDRVMREFLR-04-

RSTS00294 I----SDCRAVTIPGSSHNPHEENIAP---CLAAVSAFLR-03-

REM_190894576 V----PGCEWVLFENSSHMPHVEEKQL---CLATVSGFLS-03-

XCP_66767623 I----PDARWHVFPNSSHMPHVEEREA---CMRLVGNFLD-21-

LCA_est ADSLPSIRHNFYKSPGHHD---WNYWDS--CIHDFLDKLP-03-

LLC_estA LKKKGFDVTYESS-DGVHE---WYYWTK--KIESVLQWLP-10-

LLA_estA LKKLGFDITYESS-DGVHE---WYYWTQ--KIESVLKWLP-10-

OOE_116491533 FRKFGYEVNYQTS-HGRHE---WYYWNK--QIEKVLEWLP-10-

OOE_116490775 LRKMGYDVSYETN-PGDHE---WYYWSK--YIENILQWLP-10-

LME_116618577 LQQLNYDITYETA-PGTHE---WYYWNK--QIERVLEWLP-10-

2UZ0 LKKLGFDVTYSHS-AGTHE---WYYWEK--QLEVFLTTLP-10-

STU_55821195 LKALGLDIDYSTD-HGRHE---WYYWEK--QLEAYLEWLP-10-
