# Supplementary material for: The proteolytic system of lactic acid bacteria revisited: a genomic comparison
Source: BMC Genomics. 2010 Jan 15;11:36. doi: 10.1186/1471-2164-11-36 (PMC2827410; doi:10.1186/1471-2164-11-36)
Supplement: Additional file 3 — Superfamily tree of PepF members. The bootstrapped (n = 1000) neighbor-joining tree for PepF members in LAB [file 1471-2164-11-36-S3.PDF]

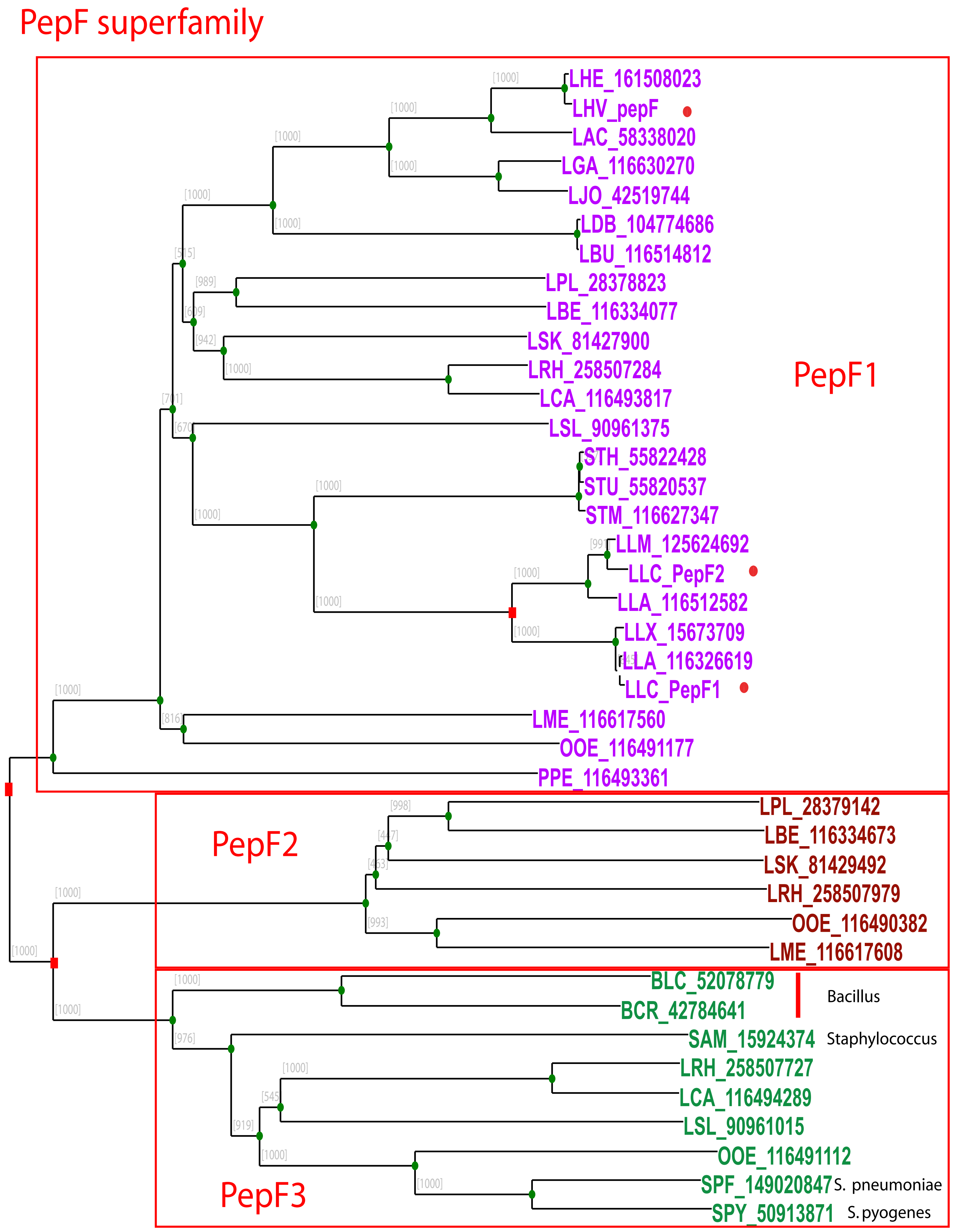

Additional File 2. Superfamily tree of PepF members. Genome abbreviations can be found in main text "Methods". For each gene, the organism abbreviations are followed by GI codes. Homologs from non-LAB strains are also included and the species/phyla are shown behind the genes. Experimentally characterized genes are highlighted by red dots. Green circles represent the speciation events, while red squares represent duplication events.
