# Supplementary material for: The proteolytic system of lactic acid bacteria revisited: a genomic comparison
Source: BMC Genomics. 2010 Jan 15;11:36. doi: 10.1186/1471-2164-11-36 (PMC2827410; doi:10.1186/1471-2164-11-36)
Supplement: Additional file 4 — Comparison of important residues of the conserved core regions and the cap region. The file contains a table describing the four structurally conserved regions and the cap regions. The identified residues within those regions, which are functionally important and/or conserved in PepI/R/L or EstA families, are highlighted [file 1471-2164-11-36-S4.DOC]

**Additional File 4**. Important residues of the core and cap regions for distinguishing the substrate specificities, as deduced from protein 3D-structure alignment.

Catalytic triad residues are highlighted in green. Residues involved in cavity forming or substrate binding are in bold.

| **residue** | **Peptidase** | | | | | **Esterase A** | | **Remarks** |
| --- | --- | --- | --- | --- | --- | --- | --- | --- |
| **nr^a^** | **1mtz** | **1azw** | **1wm1** | **PepR** | **PepI** | **2uz0** | **EstA** |  |
| **Region I (1mtz: C5-G65, 1azw: Y11-G70, 1wm1: Y14-G73, 2uz0: V2-N76)** | | | | | | | | |
| 46 | H35 | H41 | H44 | H | H | H43 | H | cavity forming residues |
| 47 | G36 | **G42** | G45 | G | G | **G44** | G | cavity in 2uz0,substrate binding 1azw |
| 48 | **G37** | **G43** | **G46** | G | G | **M45** | M | substrate binding residue in 1wm1, 1azw, oxyanion hole in 1mtz, 2uz0 |
| 49 | **P38** | P44 | **P47** | P | P | **S46** | G/S | substrate binding in 1wm1, hydrophobic pocket in 1mtz, cavity in 2uz0 |
| 50 | G39 | G45 | G48 | G | G | **G47** | G/D | cavity in 2uz0 |
| 51 | **M40** | **G46** | G49 | G | S/L/C | N48 | D/N | hydrophobic pocket in 1mtz  cavity entrance of 1azw |
| 52 | S41 | **G47** | G50 | N | T/S/P | H49 | H/E | cavity entrance of 1azw |
| 53 | H42 | **C48** | I51 | H | H | N50 | F/N | cavity entrance of 1azw |
| 54 | D43 | **N49** | S52 | E | D/N | **S51** | S/K/I/D | cavity in 2uz0, entrance of 1azw |
| 57 |  |  |  |  |  | **K54** | R/I/F | cavity in 2uz0 |
| 58 |  |  |  |  |  | **R55** | R/E/K | cavity in 2uz0 |
| **Region II (1mtz: G82-F94, 1azw: L88-G100, 1wm1: L91-G103 2uz0: Y88-K101)** | | | | | | | | |
| **Region III (1mtz: V99-L131, 1azw: W104-F136, 1wm1: W107-F139 2uz0: T114-A145)** | | | | | | | | |
| 106 | **S104** | **G109** | G112 | Q | Q | **L119** | L/M | cavity in 2uz0 |
| 107 | **S105** | **S110** | **S113** | S | S | **S120** | S | catalytic residue Ser |
| 108 | **Y106** | **W111** | **W114** | W | W | **M121** | M | oxyanion hole forming in 2uz0 and 1mtz |
| 118 | V116 | Q121 | Q124 | V/L/A | C ^b^ |  |  | Cys is important for PepI |
| 133 | **S128** | **R133** | **R136** | S | S/A |  |  | hydrophilic cavity in 1mtz, Arg for binding C-terminal of 1wm1 |
| 136 | **L131** | **F136** | **F139** | V | L/P | A145 | A/V | hydrophobic pocket in 1mtz, 1azw, 1wm1, 2uz0 |
| **Region IV (1mtz: D224-L290, 1azw: Q245-A313, 1wm1: Q247-A315, 2uz0: S180-P248)** | | | | | | | | |
| 166 | **D244** | **D266** | **D268** | E | D | **D202** | D | catalytic residue Asp/Glu |
| 167 | **E245** | **V267** | **M269** | T | L/E | **F203** | F/S | substrate binding residue in 1mtz, hydrophobic pocket forming residue in 2uz0 |
| 168 | **V246** | **V268** | A270 | M | C ^b^ |  |  | hydrophobic pocket in 1mtz, 1azw, Cys is important for PepI |
| 170 | T247 | P270 | Q272 | P | T/S | **L204** | L/I/F | hydrophobic pocket in 2uz0 |
| 198 | **H271** | **H294** | **H296** | H | H | **H231** | H | catalytic residue His;  mutated to Thr in LDE_PepL |
| 199 | **L272** | S295 | S297 | H | M | E232 | E | hydrophobic pocket in 1mtz, Met is important for PepI |
| 200 | **T273** | A296 | Y298 | H | P/S/A |  |  | hydrophilic cavity in 1mtz |
| 202 | W275 | **F297** | D299 | V/I | V/I |  |  | hydrophobic pocket in 1azw |
| 203 | E276 | E298 | E300 | D | Q/D/E | **W233** | W | cavity in 2uz0 |
| **Cap region (1mtz: S132-W223, 1azw: L137-D244, 1wm1:T140-D246, 2uz0: L146-P178)** | | | | | | | | |
|  |  | **F146** | **Y149** |  |  |  |  | hydrophobic pocket in 1wm1, 1azw |
|  | **T137** | **Y147** | **Y150** | Y | W |  |  | hydrophobic pocket in 1mtz, 1wm1 |
|  |  | **E201^c^** | **E204^c^** |  |  |  |  | substrate binding in 1wm1, 1azw |
|  | **Y205** | **F226** | **F228** | Y | Y |  |  | substrate binding in 1mtz, hydrophobic pocket in 1azw |
|  | **N209** | **E230** | **E232** | Q | W ^b^ |  |  | hydrophobic pocket in 1mtz, substrate binding in 1wm1,1azw |
|  | G210 |  |  | G | G |  |  | cavity forming in peptidase |
|  | P211 |  |  | D | P |  |  | cavity forming in peptidase |
|  | **N212** |  |  | N | N ^b^ |  |  | entrance cavity in 1mtz |
|  | **E213 ^c^** | **F234** | **F236** | E | E |  |  | substrate binding in 1mtz ^d^ hydrophobic pocket in 1wm1 |
|  |  |  |  |  |  | **L158** | M/L/T | hydrophobic pocket in 2uz0 |
|  |  |  |  |  |  | **W164** | W/L | hydrophobic pocket in 2uz0 |

1. Position in the multiple sequence alignment of the core regions, see Additional File 3;
2. Conserved in LAB strains
3. 1azw and 1mtz have a Glu-residue, whose side-chain is near the 3 active site residues. However these Glu side chains are coming from a different part of the sequence, so they do not align in the multiple sequence alignment (1azw:Glu201, 1mtz:Glu213, see Figure 4b). This residue is possibly involved in substrate binding. 1uz0 has no such Glu residue.
4. This substrate binding residue is conserved as Glu, except in OOE_116490346 where it is mutated into Pro.
