# Supplementary material for: The proteolytic system of lactic acid bacteria revisited: a genomic comparison
Source: BMC Genomics. 2010 Jan 15;11:36. doi: 10.1186/1471-2164-11-36 (PMC2827410; doi:10.1186/1471-2164-11-36)
Supplement: Additional file 5 — PepX superfamily tree. The file contains a bootstrapped (n = 1000) NJ tree for PepX family homologs of LAB [file 1471-2164-11-36-S5.PDF]

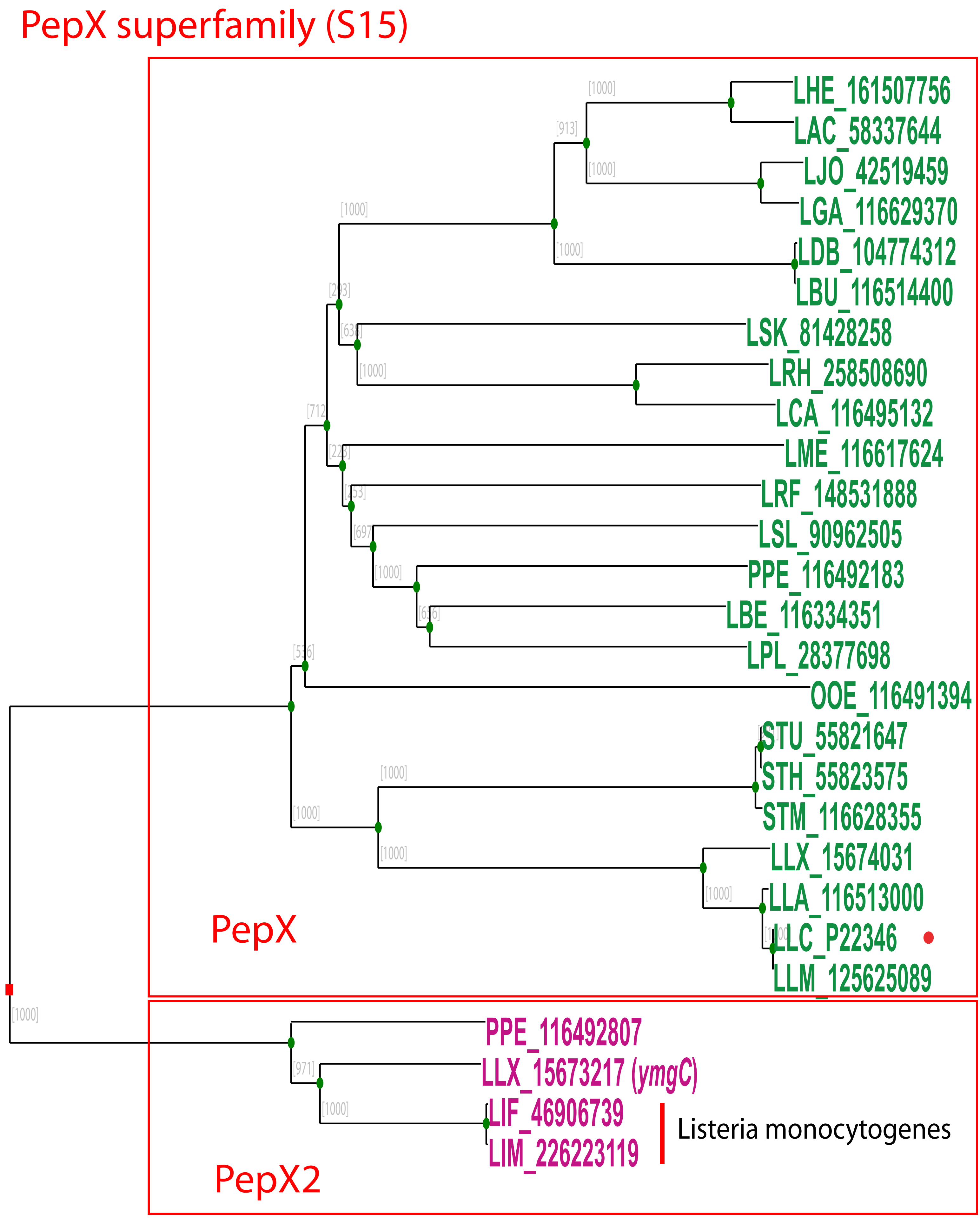

Additional File 5. PepX superfamily tree. For each gene, the organism abbreviations are followed by GI codes. Homologs from *Listeria monocytogenes* are highlighted. Experimentally verified PepX from *L. lactis* is indicted by a red dot. Green circles represent the speciation events, while red squares represent duplication events.
